# Multisegmental spinal arteriovenous malformation associated with the Parkes–Weber syndrome: A case report and literature review

**DOI:** 10.1097/MD.0000000000042832

**Published:** 2025-06-20

**Authors:** Li Tao, Xian Zhang, Jian Xue

**Affiliations:** a Department of Radiology, The First Affiliated Hospital of Chongqing Medical University, Chongqing, China; b Department of Hospital Infection, The Third Affiliated Hospital of Zunyi Medical University, The First People’s Hospital of Zunyi, Guizhou, China; c Department of Health Management, Zunyi Medical and Pharmaceutical College, Guizhou, China.

**Keywords:** case description, contrast-enhanced magnetic resonance angiography, Parkes– Weber syndrome, spinal arteriovenous malformation

## Abstract

**Rationale:**

Spinal arteriovenous malformation (SAVM) and Parkes–Weber syndrome (PWS) are both rare vascular lesions, and SAVM associated with PWS is even rarer. In the last 2 decades, about 9 cases have been reported, making this case noteworthy.

**Patients concerns:**

A 33-year-old man presented to the emergency department with a complaint of palpitations that persisted unrelieved after going to the bathroom. During hospitalization, the patient was diagnosed with atrial fibrillation and multisegmental SAVM associated with PWS by the imaging findings.

**Diagnosis:**

Multisegmental SAVM associated with PWS.

**Intervention:**

Embolization for SAVM and compression stockings for PWS.

**Outcome:**

A significant improvement of the abnormal connections between the arteries and veins in the SAVM.

**Lessons:**

Both SAVM and PWS have vascular malformations. It is important to diagnose them early and accurately by appropriate screening methods.

## 1. Introduction

Parkes–Weber syndrome (PWS) is a rare congenital vascular anomaly after British dermatologist Frederick Parkes Weber, who first described the syndrome in 1907, characterized by capillary, venous, and lymphatic malformations in conjunction with arteriovenous fistulas (AVFs).^[1]^ It is often associated with hypertrophy of 1 limb due to the extensive involvement of blood vessels. Although the syndrome primarily affects the extremities, there are instances where these vascular anomalies extend to other regions, including the spine.^[2–5]^ Spinal arteriovenous malformation (SAVM) are abnormal connections between the arteries and veins in the spinal cord, which can lead to a range of neurological deficits due to altered blood flow and potential hemorrhage.^[6]^ The association between SAVM and PWS is extremely rare, making it a subject of significant clinical interest.

In this study, we reported a rare case of multisegmental SAVM associated with PWS in a patient, highlighting the imaging findings, management, and the follow-up results. This report provides a comprehensive review of this association around the world over the last 20 years and aims to emphasize the importance of recognizing this rare association and to call for the application of more appropriate screening tools to facilitate early diagnosis and thus improve patient prognosis.

## 2. Case information

### 2.1. Chief complaints

On January 12, 2021, a 33-year-old male presented to our emergency department with sudden onset of palpitations that persisted unrelieved after going to the bathroom, accompanied by chest tightness and black haze.

### 2.2. History of present illness

The patient first diagnosed atrial fibrillation (AF) on October 2, 2019, which resumed rate 2 weeks later via oral amiodarone. He had no further episodes of AF before being admitted to our hospital on January 12, 2021.

### 2.3. History of past illness

**Figure 1.**
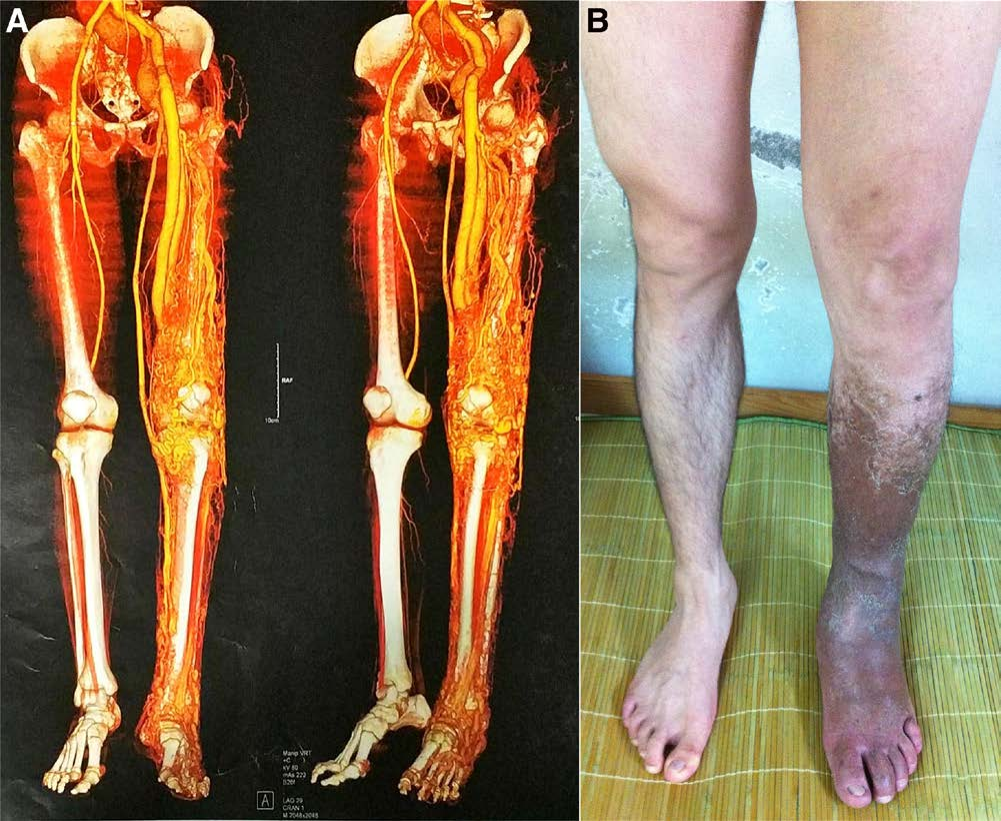
A 33-yr-old male with multisegmental spinal arteriovenous malformation and PWS. (A) The computed tomography angiography of both lower extremities of the patient. (B) Localized tissue overgrowth and localized skin pigmentation in patients. PWS = Parkes–Weber syndrome.

In 2006, computed tomography angiography of his lower limb arteries was performed in an outside hospital, which showed arteriovenous fistula, and embolization was performed. In 2012, an aneurysm of the left femoral artery was found and observed; in 2016, it was found to have increased in size, and he underwent aneurysm resection (Fig. 1A). He was diagnosed with gout at an outside hospital in 2019. PWS combined with multisegmental SAVM was diagnosed at an outside hospital, and SAVM embolization was performed in May 2020 and November 2020.

### 2.4. Personal and family history

The patient’s mother had hypertrophic cardiomyopathy.

### 2.5. Physical examination

The patient had numbness in both lower limbs. His left lower limb is about 3 cm longer than the right lower limb, with high skin temperature and calf pigmentation (Fig. 1B). The patient had an average ventricular rate of 115 beats/min.

### 2.6. Laboratory examinations

The electrocardiogram showed left ventricular hypervoltage occasionally accompanied by intraventricular differentiated conduction. The plasma B-type natriuretic peptide level was 4327 ng/L (reference range: 0–97 ng/L) (Fig. S1, Supplemental Digital Content, https://links.lww.com/MD/P255).

### 2.7. Imaging examinations

**Figure 2.**
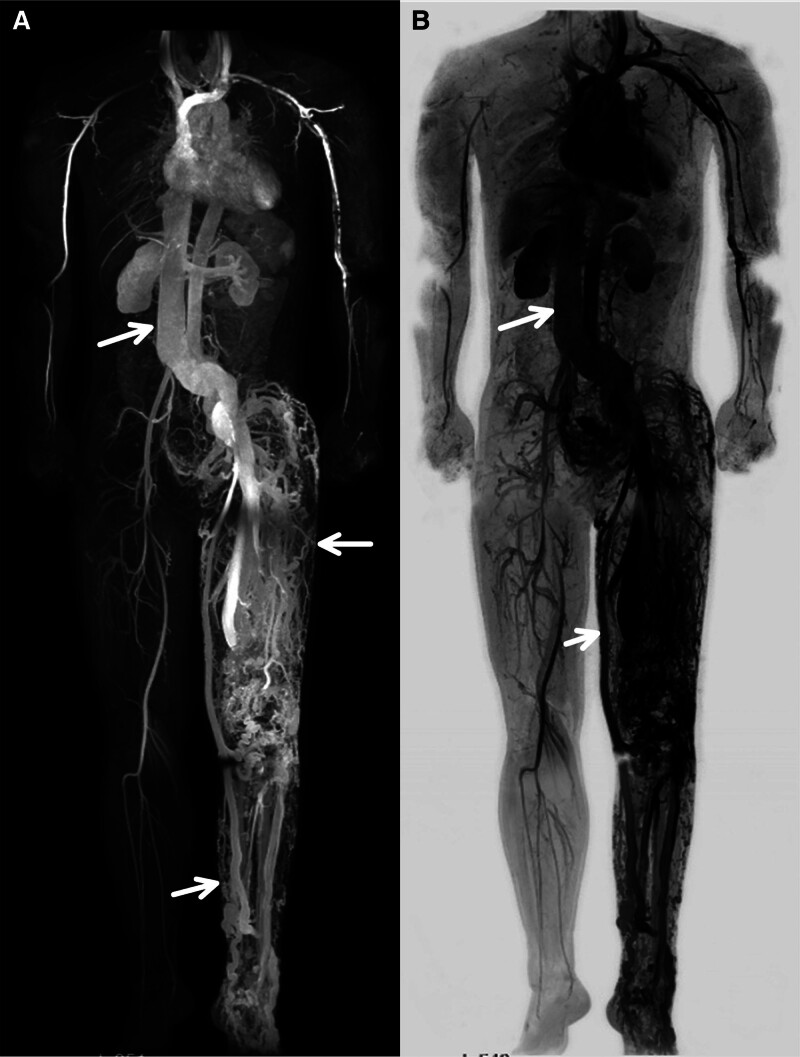
The patient underwent whole-body angiography using modified DIXON technique combined with CE-MRA and bolus track technique. (A) CE-MRA showed marked dilation of the inferior vena cava compared to the abdominal aorta, involving the left iliac vein, with dilation of both arteries and veins in the left lower extremity. (B) CE-MRA showed dilation of the left great saphenous vein and arteriovenous malformations in the left lower. CE-MRA = contrast-enhanced magnetic resonance angiography.

**Figure 3.**
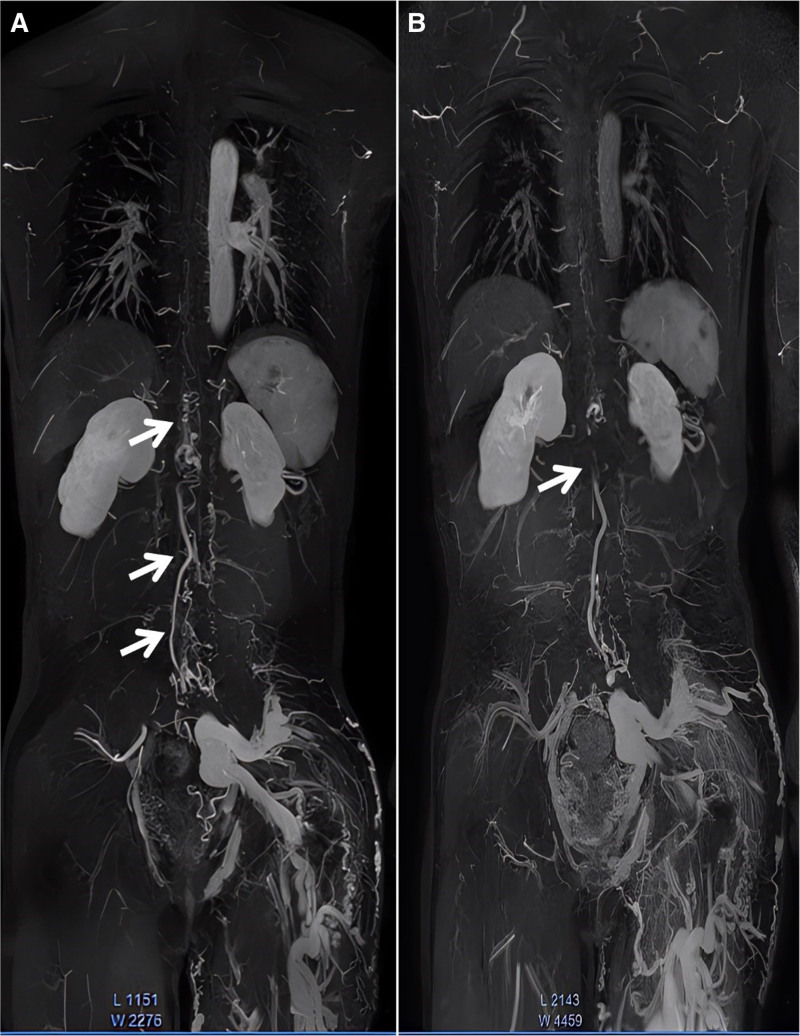
(A) CE-MRA showing spinal arteriovenous malformation (T9–L4) before embolization. (B) CE-MRA showed significant improvement of the abnormal connections between the arteries and veins in the SAVM after embolization (T12–L1). CE-MRA = contrast-enhanced magnetic resonance angiography, SAVM = spinal arteriovenous malformation.

The patient underwent whole-body angiography using a Philips Ingenia 3.0T digital network architecture magnetic resonance system with a modified DIXON technique combined with contrast-enhanced magnetic resonance angiography (CE-MRA) and a bolus track technique on January 19, 2021, and October 8, 2022, respectively. on January 19, 2021, CE-MRA showed that during the arterial phase, the inferior vena cava was opacified and larger in caliber than the abdominal aorta (Fig. 2A), and that the veins of the left lower extremity were markedly dilated compared to those of the right lower extremity, consistent with arteriovenous malformation of the left lower limb (Fig. 2B). On October 8, 2022, the results showed significant improvement of the abnormal connections between the arteries and veins in the SAVM after embolization (T12–L1) (Fig. 3A and B).

### 2.8. Final diagnosis

The patient presented with symptoms primarily attributable to AF, while further investigation revealed the presence of multisegmental SAVM associated with PWS.

### 2.9. Treatment

The patient’s current AF was a second recurrence. In the case of unsuccessful rate restoration by intravenous amiodarone, no other measures were taken because the patient’s whole heart was significantly enlarged and was highly susceptible to postoperative recurrence if either electrical cardioversion or radiofrequency ablation was performed. After stabilization of the heart rate, the patient was discharged from the hospital on long-term oral metoprolol (47.5 mg/bid) for ventricular rate control, oral rivaroxaban (15 mg/d) for anticoagulation, oral sacubitril valsartan sodium (100 mg/bid) for anti-heart failure, and oral febuxostat (20 mg/d) for gout and hyperuricemia.

### 2.10. Outcome and follow-up

Our follow-up period was from October 2022 to October 2023. The development of the different diseases in this patient during the follow-up period was as follows:

#### 2.10.1. Atrial fibrillation

During follow-up, the patient developed permanent AF due to the enlargement of his entire heart. After a comprehensive analysis, the cardiologist decided to maintain his current medications to control ventricular rate and prevent stroke.

#### 2.10.2. Parkes–Weber syndrome

During the follow-up period, the patient used a compression garment for daily care to help reduce pain and swelling and to protect the limb from impacts and scrapes, which could cause bleeding.

#### 2.10.3. Spinal arteriovenous malformation

During the follow-up period, the patient’s buttock numbness due to his spinal vascular malformation did not worsen, and claudication decreased.

## 3. Discussion

SAVM and PWS are distinct vascular disorders with different etiologies and demographics.

SAVM and PWS are both rare disorders. Most of the available reports are of cases scattered all over the world, so there is no accurate incidence yet. SAVM is characterized by abnormal tangling of spinal cord blood vessels, which disrupts normal blood flow and can lead to a variety of neurological symptoms.^[7]^ PWS is characterized by an arteriovenous malformation (AVM) of the affected limbs as well as overgrowth of the soft tissues and bones.^[8]^

SAVM and PWS are often scattered sporadically. The pathogeneses of these 2 conditions are associated with abnormalities in vascular development. The mutations in *RASA 1* gene may lead to PWS, whereas the exact etiology of SAVM is not fully understood.^[9–11]^ Demographically, SAVM can affect people of any age but is rare in young people.^[12]^ PWS is usually diagnosed in childhood or early adolescence.^[13,14]^ The incidence of these 2 disorders may vary in different populations.

Both SAVM and PWS are clinically rare, so SAVM associated with PWS is even rarer. Using the keywords “spinal arteriovenous malformation” and “PWS,” a search was conducted for relevant literature published in the PubMed and Web of Science medical databases in the past 20 years. A total of 9 cases were found (Table 1). Among these 9 patients, 5 were females and 4 were males, indicating no gender difference in incidence. The median age of the 9 patients was 14 years old. Among the 9 patients, the affected spinal cord segments were mainly thoracic, lumbar, and conus medullaris.

**Table 1:** Summary of reported cases of SAVM associated with Parkes–Weber syndrome.

Report no.	Sex/age of patients	Affected spinal cord segment	Symptoms of SAVM	Symptoms of PWS	PWS segment	Treatment of SAVM	Treatment for PWS	Outcome	Reference
1	M/10 yr	L2–L5	Cardiac insufficiency	Hypertrophic right leg, cutaneous capillary malformation and multiple fistulas	Right leg	Embolization	NS	Recovery	^[2]^
2	F/24 yr	Conus medullaris	Repetitive SAH	Hypertrophic left leg, port-wine stain, varicose veins and arteriovenous fistulas	Left leg	Embolization	Without any active treatment	Recovery	^^[15]^^
3	F/3 yr	L2	NS	Hypertrophic right leg, multiple capillary malformations	Right leg	Embolization	NS	NS	^[3]^
4	F/14 yr	Conus medullaris	SAH	Hypertrophic left leg, multiple capillary malformations,	Left leg, left breast	Surgical resection and embolization	NS	Recovery	^[16]^
5	F/12 yr	T12–L4	Back pain, myelopathy	Hypertrophic left leg, varicose veins and extensive fistulas	Left leg	Embolization	Embolization	Amputation left foot	^[4]^
	M/24 yr	T8–L4	Repetitive SAH, myelopathy	Hypertrophic right leg, varicose veins and multiple fistulas	Right leg	Embolization	NS	Paraplegia	
6	F/17 yr	T12–L1	A vascular malformation associated with intraspinal hemorrhage	Hypertrophic right leg, varicose veins, multiple arteriovenous fistulas and skin pigmentation	Right leg	Surgical resection and embolization	NS	Significantly improved	^[5]^
7	M/9 mo	NS	Progressive weakness in the lower limbs, evolving to paraplegia	Hypertrophic left leg, port-wine stain, fistulas	Left leg	Embolization	NS	Recovery	^[17]^
8	M/33 yr	T9–L4	Progressive weakness in the lower limbs	Hypertrophic left leg, varicose veins, multiple arteriovenous fistulas and skin pigmentation	Left leg	Embolization	NS	Significantly improved	This study

NS = not stated, PWS = Parkes–Weber syndrome, SAH = subarachnoid haemorrhage, SAVM = spinal arteriovenous malformation.

According to the literature review presented in Table 1, the typical clinical findings of PWS is 1 hypertrophic leg, varicose veins, and multiple AVFs. In contrast, SAVM presents with progressive neurologic symptoms, such as weakness, numbness, or tingling in the limbs caused by hemorrhage from malformed blood vessels and myelopathy.

Based on the presence of vascular malformations in both SAVM and PWS, imaging findings will show abnormal blood flow patterns characteristic of AVM, for example, snake-like vessels on the cord’s surface or abnormal connections among the arteriovenous vessels.

Treatment of SAVM includes endovascular embolization to block the malformed vessel and reduce its blood supply, open surgery to remove the AVM and restore normal blood flow to the spinal cord, and stereotactic radiation therapy to constrict or seal the malformed vessel.^[18,19]^ The prognosis for patients with SAVM depends on the size and location of the malformed vessel, the degree of neurologic deficit, and the efficacy of treatment. As shown in the literature review in Table 1, with timely and appropriate intervention, many patients can achieve symptomatic improvement and neurological stabilization. However, untreated or poorly managed SAVM can lead to progressive neurologic deterioration.

Treatment for PWS includes endovascular embolization to block the abnormal blood vessels, reducing blood flow and relieving symptoms, open surgery to remove the abnormal blood vessels, laser therapy to reduce port-wine stains, and long-term compression garments to reduce pain and swelling.^[13]^ PWS is a progressive disease, and with continued medical care or intervention, the prognosis is generally good, with many people experiencing relief of symptoms and an improved quality of life.

SAVM is easily confused with degenerative spinal lesions, spinal cord tumors, spinal cord cavities, and myelitis, leading to mis- and underdiagnosis (Table 2). In contrast to SAVM, degenerative spinal lesions are characterized by structural changes, such as intervertebral disc degeneration, vertebral body deformity, and osteophytes. Spinal cord tumors often exhibit more evident local masses and signs of nerve root compression. Syringomyelia usually presents on imaging as cystic lesions without obvious vascular structures. Spinal cord inflammation usually has inflammatory imaging manifestations, such as growing lesions on contrast-enhanced magnetic resonance imaging.

**Table 2:** Differential diagnosis table for SAVM.

Differential disease	Etiology	Systemic findings	Imaging findings
SAVM	Abnormal vascular structures within the spinal cord; congenital or acquired	Motor or sensory impairments, pain, or paralysis	Abnormal vascular structures and their relationship to surrounding tissues
Degenerative spinal lesions	Aging; acquired	Back pain, limited mobility, stiffness, and neurological deficits	Disc protrusion, disc degeneration, and spinal osteophytes
Spinal cord tumors	Primary or metastatic	Neurological deficits, spinal cord compression symptoms, and pain	The size, location, and characteristics of the tumor
Spinal cord cavities	Syringomyelia, trauma, or infection; congenital or acquired	Sensory disturbances, motor deficits, and pain	The cavity within the spinal cord and its relationship to surrounding structures
Myelitis	Infections, autoimmune diseases, or other inflammatory conditions affecting the spinal cord	Fever, headache, fatigue, and neurological symptoms	Inflammatory changes in the spinal cord

SAVM = spinal arteriovenous malformation.

PWS is most easily confused with Klippel–Trénaunay syndrome.^[8]^ However, PWS occurs because of vascular malformations, whereas Klippel–Trénaunay syndrome is a disorder in which blood vessels and/or lymphatic vessels do not form properly. In addition, patients with PWS present with both AVM and AVFs with limb hypertrophy.

In summary, both of SAVM and PWS present vascular malformations. Malformed vessels are prone to rupture and bleeding, and multiple AVFs may lead to heart failure. Therefore, early recognition and diagnosis of these specific vascular pathologies is important. The modified DIXON CE-MRA has no ionizing radiation, uses very little contrast agent, uses breath-hold rapid scanning to achieve whole-body vascular imaging, and does not require subtraction, with excellent image quality. It is an ideal screening tool for vascular abnormality examination.

## Author contributions

**Conceptualization:** Jian Xue.

**Data curation:** Xian Zhang.

**Funding acquisition:** Jian Xue.

**Investigation:** Li Tao.

**Writing – original draft:** Li Tao.

**Writing – review & editing:** Jian Xue.

## Supplementary Material


