# Supplementary figures and images for: Multisegmental spinal arteriovenous malformation associated with the Parkes–Weber syndrome: A case report and literature review
Source: Medicine (Baltimore). 2025 Jun 20;104(25):e42832. doi: 10.1097/MD.0000000000042832 (PMC12187294; doi:10.1097/MD.0000000000042832)

Figure S1


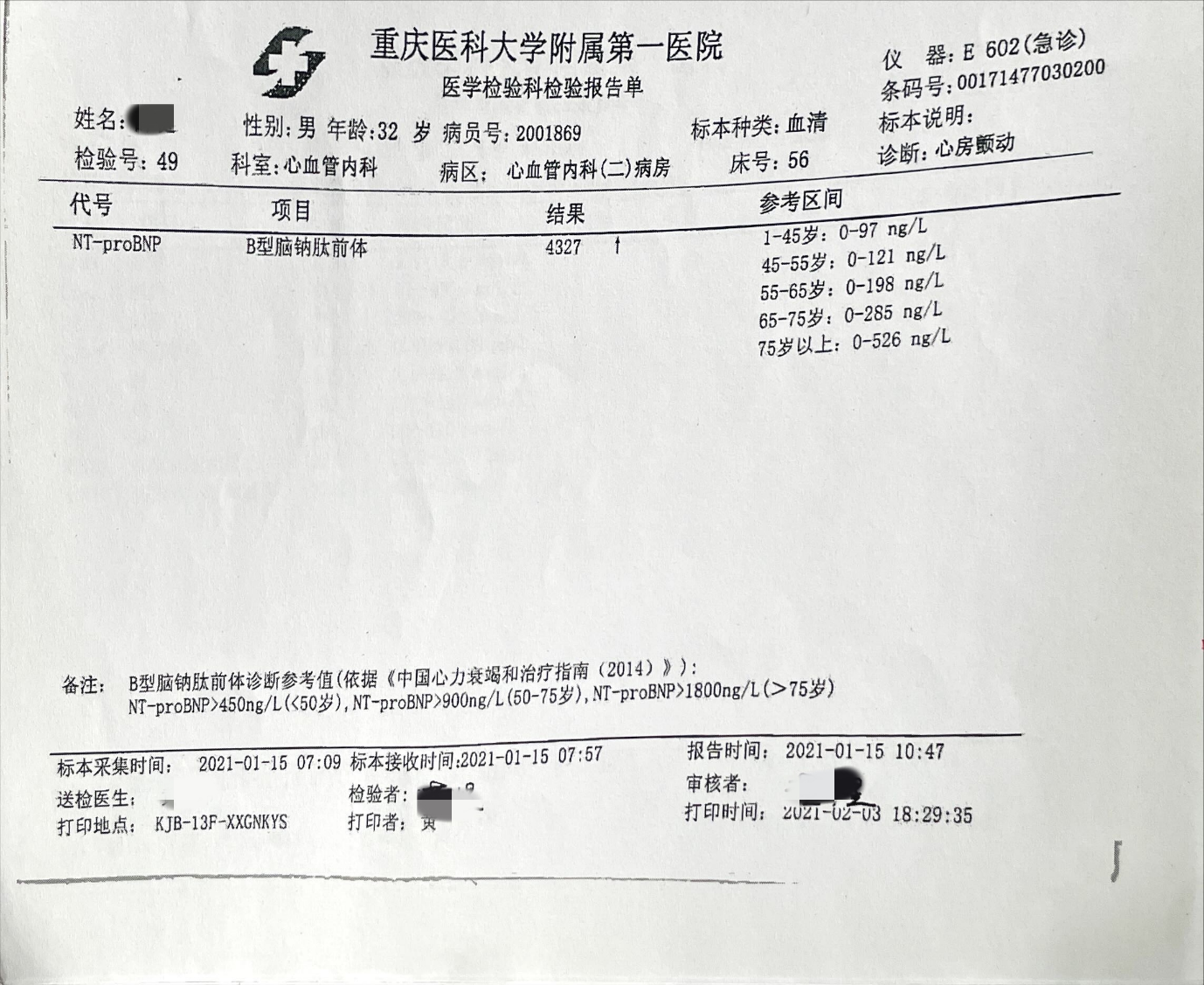

Supplement: Supplementary file 1 [file medi-104-e42832-s001.docx]
